# Prevalence of Viral and Bacterial Co-Infections in SARS-CoV-2-Positive Individuals in Cyprus 2020–2022

**DOI:** 10.3390/biomedicines13051236

**Published:** 2025-05-19

**Authors:** George Krashias, Christina Tryfonos, Stavros Bashiardes, Jan Richter, Christina Christodoulou

**Affiliations:** 1 Department of Molecular Virology, Cyprus Institute of Neurology and Genetics, 2371 Nicosia, Cyprus; georgek@cing.ac.cy (G.K.); tryfonos@cing.ac.cy (C.T.); sbash@cing.ac.cy (S.B.); cchristo@cing.ac.cy (C.C.); 2 Postgraduate School, Cyprus Institute of Neurology and Genetics, 2371 Nicosia, Cyprus

**Keywords:** COVID-19, SARS-CoV-2, co-infections, respiratory viruses, bacteria

## Abstract

The COVID-19 pandemic has had a profound impact on healthcare systems worldwide, with severe consequences on the global economy and society. The clinical presentation of SARS-CoV-2 infection varies widely, ranging from asymptomatic cases to severe disease and death. Coinfection with other respiratory pathogens in SARS-CoV-2-positive individuals may exacerbate symptom severity and lead to poorer clinical outcomes. **Background/Objectives**: This study is the first to investigate the prevalence of viral and bacterial co-infections in SARS-CoV-2-positive individuals in Cyprus. **Methods**: A total of 1111 SARS-CoV-2-positive nasopharyngeal swab samples collected from non-hospitalized patients were analyzed for the presence of 18 viral and 3 bacterial respiratory pathogens. **Results**: Of these, 51 samples (4.6%) were found to have at least one additional respiratory pathogen. The most frequently detected viruses were rhinovirus/enterovirus (*n* = 28; 2.5%) and adenovirus (*n* = 8; 0.7%), while the bacterial pathogens identified were *Legionella pneumophila* (*n* = 1; 0.1%) and *Bordetella pertussis* (*n* = 1; 0.1%). The highest proportion of co-infections was observed in the youngest age group (<10 years), where 52.9% of co-infections were identified, followed by the 30–39 age group, which accounted for 15.7% of cases. Among single respiratory virus co-infections, rhinovirus/enterovirus (27.5%) and adenovirus (13.7%) were the most frequently detected in the <10 age group, followed by RSV (3.9%), bocavirus, influenza B, HMPV A + B, and coronavirus NL63 (each at 2%). **Conclusions**: The current study underscores the importance of simultaneous testing for SARS-CoV-2 and other respiratory pathogens, as this may have significant implications for both individual patient care and healthcare services.

## 1. Introduction

Severe acute respiratory coronavirus 2 (SARS-CoV-2) is responsible for coronavirus disease 2019 (COVID-19), already having caused a loss of 7 million lives globally (https://covid19.who.int/, accessed on 16 February 2025). SARS-CoV-2 is transmitted via aerosol droplets, and it mainly infects the respiratory tract [1]. Infected patients experience symptoms ranging from mild illness up to a critical care condition, which requires hospitalization at intensive care units. The common symptoms of COVID-19 include fever, cough, dyspnea, fatigue, myalgia, and diarrhea [2].

Clinical symptoms of COVID-19 resemble those of other respiratory illnesses, caused by other viruses and bacteria [3,4]. Of equal importance, co-infections with other respiratory pathogens may lead to greater severity of illness, complicating treatments targeting SARS-CoV-2 or the co-pathogen. For instance, influenza infection in COVID-19 patients has been associated with increased hospitalization, disease symptoms, and mortality rate [5,6,7]. Similarly, bacterial co-infection in COVID-19 appears to be a major risk factor for mortality, ICU admission, and mechanical ventilation, and can result from both superinfections and co-infections with other microbial pathogens [8].

Co-infection with viral and bacterial pathogens has been examined in numerous studies, most of which have focused on hospitalized patients who are at increased risk of complications [9,10,11]. Among the most frequently detected viruses in SARS-CoV-2-infected patients are influenza A, influenza B, respiratory syncytial virus (RSV), and rhinovirus. Bacterial co-infections commonly include pathogens such as *Pseudmonas aeruginosa*, *Haemophilus influenzae*, and *Mycoplasma pneumonia*, while fungal co-infections—most notably with *Aspergillus*—have also been reported [12]. In contrast, co-infections among outpatients have been less extensively studied [13,14]. Nevertheless, these co-infections may still contribute to the transmission of respiratory pathogens in the community and have significant implications for public health and healthcare services.

As a result of viral and bacterial co-infections, COVID-19 may be overlooked and thereby diagnosed late, hindering the prompt administration of designated antiviral therapies. This in turn is expected to negatively impact clinical progression of the disease. Therefore, data on the prevalence and the most common co-infecting viral and bacterial pathogens will help clinicians and health agencies to treat patients and implement appropriate infection control measures, respectively [9]. Equally important, co-infections in outpatients may alter respiratory transmission in the community, increasing exposure among vulnerable groups, including children and the elderly.

SARS-CoV-2 was detected in Cyprus for the first time on 9 March 2020. The Republic of Cyprus is one of the countries in Europe least affected by the COVID-19 pandemic, presumably due to its rapid and effective response strategy, which included a high number of COVID-19 tests, effective tracing and isolation of cases and their contacts, together with preventive measures (e.g., social distancing, wearing face masks, and hand washing). In addition, being an island guaranteed high effectiveness of airport closure with regard to the importing of new cases [15,16].

Up-to-date data regarding co-infection with other respiratory microorganisms in SARS-CoV-2-positive patients in Cyprus is still lacking. Therefore, the present study was conducted in an attempt to determine the frequency of respiratory viral and bacterial co-infections among SARS-CoV-2-positive nasopharyngeal swab (NPS) samples of non-hospitalized patients. The present study is expected to provide previously unavailable data, underscoring the importance of continuing surveillance programs for various respiratory pathogens.

## 2. Materials and Methods

### 2.1. Sample Selection

**Figure 1:**
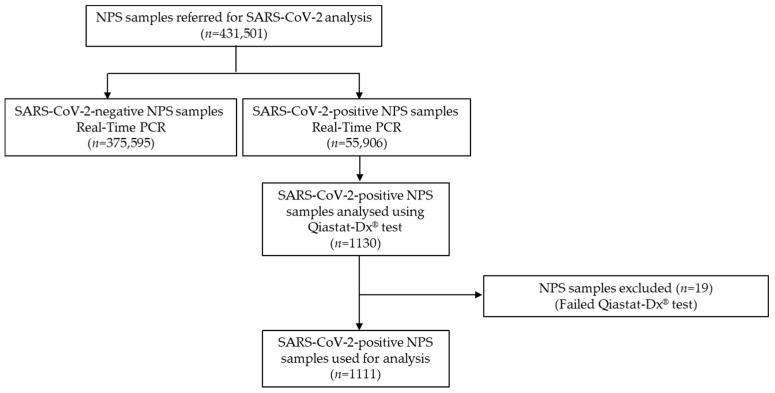
Study flow chart.

**Figure 2:**
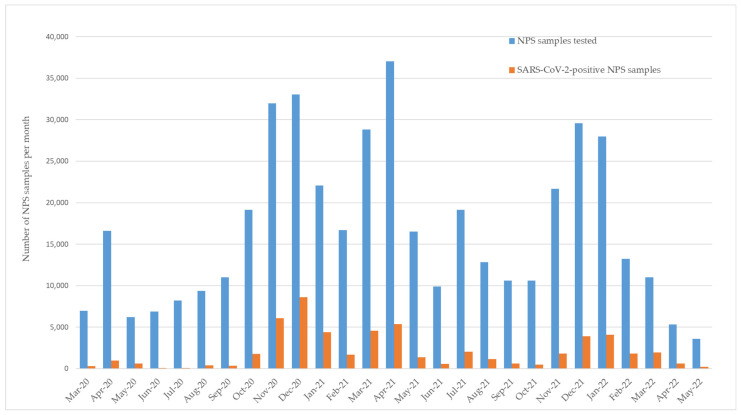
Monthly number of NPS samples analyzed for SARS-CoV-2 and number of SARS-CoV-2-positive NPS samples identified between March 2020 and May 2022. NPS: nasopharyngeal swab.

The Department of Molecular Virology at the Cyprus Institute of Neurology and Genetics serves as the reference laboratory for SARS-CoV-2, as designated by the Ministry of Health of the Republic of Cyprus. During the COVID-19 pandemic, the Department received NPS samples as part of a national effort led by the Ministry of Health to monitor SARS-CoV-2 circulation within the community. NPS samples were collected from non-hospitalized individuals identified as close contacts of confirmed COVID-19 cases, defined as individuals who had face-to face contact within two meters for more than 15 min, or who had physical or unprotected contact with a confirmed case in a closed environment [17]. Importantly, the analyzed samples did not represent nosocomial (hospital-acquired) infections, as all NPS samples were collected outside of a hospital setting. For this retrospective observational study, 1111 SARS-CoV-2-positive NPS samples were selected for further analysis (518 males, 593 females; age range: newborn to 92 years old), corresponding to approximately 2% of SARS-CoV-2-positive cases identified each month during the study period. A detailed description of the sample selection algorithm is provided in Figure 1. NPS samples analyzed each month were proportional to the percentage of SARS-CoV-2-positive NPS samples for that specific month. In Figure 2, the monthly distribution of the number of samples analyzed, as well as the number of SARS-CoV-2-positive NPS samples identified, is shown. NPS samples were analyzed for their SARS-CoV-2 status using a qRT-PCR assay, as previously described [15].

### 2.2. QIAstat-Dx^®^ Respiratory SARS-CoV-2 Panel

QIAstat-Dx^®^ Respiratory SARS-CoV-2 Panel was used according to the manufacturer’s instructions, as previously described [18]. Briefly, 300 µL of NPS sample was loaded manually into the single-use QIAstat-Dx^®^ Respiratory SARS-CoV-2 Panel cartridge (QIAGEN GmbH, QIAGEN Strasse 1, D-40724 Hilden, Germany) and set on a QIAstat-Dx^®^ Analyser (QIAGEN GmbH, QIAGEN Strasse 1, D-40724 Hilden, Germany) for the detection of the following viral and bacterial microorganisms: adenovirus, bocavirus, coronavirus 229E, coronavirus HKU1, coronavirus NL63, coronavirus OC43, human metapneumovirus A/B, influenza A, influenza A H1, influenza A H1N1/pdm09, influenza A H3, influenza B, parainfluenza virus 1, parainfluenza virus 2, parainfluenza virus 3, parainfluenza virus 4, respiratory syncytial virus A/B, rhinovirus/enterovirus, SARS-CoV-2, *Bordetella pertussis*, *Legionella pneumophilia*, and *Mycoplasma pneumoniae*. The presence of SARS-CoV-2 and 21 other respiratory pathogens was determined, and cycle threshold values were obtained if SARS-CoV-2 and/or other respiratory pathogens were detected [18,19].

### 2.3. Statistical Analysis

The prevalence of co-infections was calculated and expressed as both absolute numbers and percentages for the overall cohort as well as for relevant subgroups. To evaluate differences in co-infection rates between males and females, Fisher’s exact test was used, with a *p*-value of <0.05 considered statistically significant. Additionally, to assess whether a statistically significant trend existed in co-infection rates across different age groups and years, a chi-square test for the trend was performed. A *p*-value of <0.05 was considered indicative of a significant association between age and the likelihood of co-infection. All statistical analysis was performed using GraphPad Prism 5.02 for Windows (GraphPad Software, La Jolla, CA, USA).

## 3. Results

### 3.1. Co-Infection of Respiratory Pathogens with SARS-CoV-2

**Figure 3:**
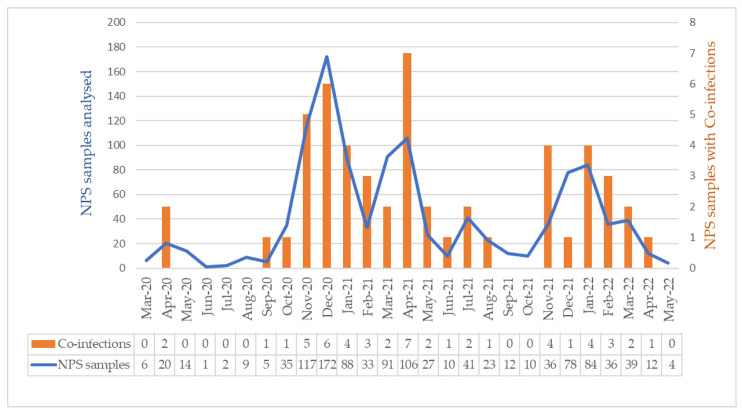
Temporal distribution of NPSs analyzed and co-infections with respiratory viruses and bacteria during the study period. Note that the total number of samples analyzed is shown on the left *y*-axis (blue graph; scale 0–200), and the number of co-infections is shown on the right *y*-axis (orange graph; scale 0–8). NPS: nasopharyngeal swab.

A total of 1111 SARS-CoV-2-positive NPS were included in the analysis. The demographic characteristics of the study participants are presented in Table 1. During the study period, each new wave of SARS-CoV-2 was associated with the emergence of a new variant of concern. Overall, it was observed that the prevalence of co-infections was proportional to the number of samples analyzed (Figure 3).

The annual distribution of co-infections and respiratory pathogens detected during the study period is shown in Table 2 and Table 3. Among the 1111 SARS-CoV-2-positive NPS samples, 51 samples (4.6%) were identified with at least one additional respiratory pathogen (Table 2). Briefly, 47 samples (4.1%) and 2 samples (0.2%) were identified with an additional one and two viruses other than SARS-CoV2, respectively. Two samples (0.2%) were identified to be co-infected with bacteria (Table 2). Although a marginal increase in co-infection rates was observed over the years, the trend was not statistically significant (*p* > 0.05, χ^2^; Table 2).

The most frequently detected viruses were rhinovirus/enterovirus (*n* = 28; 2.5%) and adenovirus (*n* = 8; 0.7%) (Table 3). Less frequently, we observed co-infection with RSV (*n* = 4; 0.4%), InfB (*n* = 3; 0.3%), human metapneumovirus A/B (*n* = 2; 0.2%), coronavirus NL63 (*n* = 2; 0.2%), coronavirus OC43 (*n* = 2; 0.2%), and bocavirus (*n* = 2; 0.2%) (Table 3). A co-infection with a bacterial pathogen was observed in two samples from the year 2020 (*L. pneumophila* and *B. pertussis*), whereas co-infection with two additional viruses was observed in only two samples in year 2021 (coronavirus NL63 and rhinovirus/enterovirus; coronavirus OC43 and RSV) (Table 3). All samples were found to be negative for coronavirus 229E, coronavirus HKU1, influenza A, influenza A H1, influenza A H1N1/pdm09, influenza A H3, parainfluenza virus 1, parainfluenza virus 2, parainfluenza virus 3, parainfluenza virus 4, and Mycoplasma pneumoniae. The rate of males to females in the coinfection cases was 28:23, with males being 1.22 times more likely to be infected with at least one additional respiratory pathogen (odds ratio: 1.416; lower 95% CI: 0.806; higher 95% CI: 2.489). Nevertheless, this difference did not reach statistical significance (*p* > 0.05), suggesting that both males and females are equally susceptible to co-infections with respiratory pathogens.

**Figure 4:**
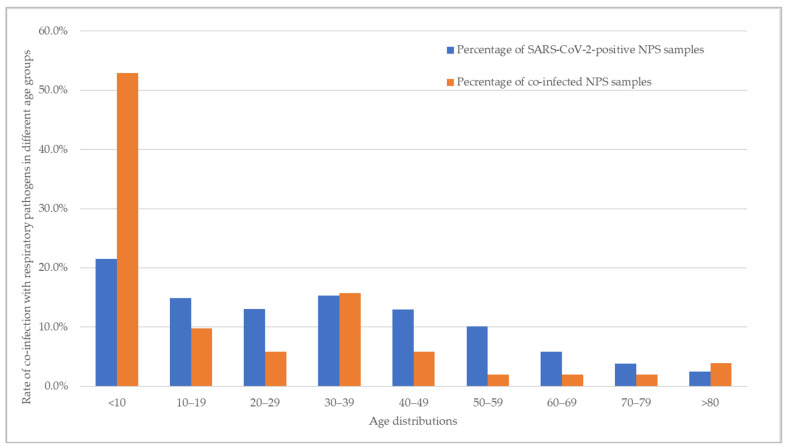
The age distribution of NPS samples analyzed (*n* = 1111) and rate of respiratory pathogens co-infection cases among the study subjects with co-infection (*n* = 51).

The age distribution of NPSs analyzed, as well as respiratory pathogen co-infection cases among the study subjects with co-infection, can be seen in Figure 4. Overall, the prevalence of co-infections appeared to be age-dependent, showing a decreasing trend with increasing age (*p* = 0.0003, χ^2^). The highest proportion of co-infections was observed in the youngest age group <10 years, followed by the age group 30–39, with 52.9% and 15.7% of co-infections with a respiratory pathogen other than SARS-CoV-2, respectively, detected in these age groups. The remaining co-infections detected in order of decreasing prevalence were age groups 10–19 (9.8%), 20–29 and 40–49 (both at 5.9%), >80 (3.9%), and 50–79 (2.0%).

### 3.2. Variation in Co-Infection with Age Group

**Figure 5:**
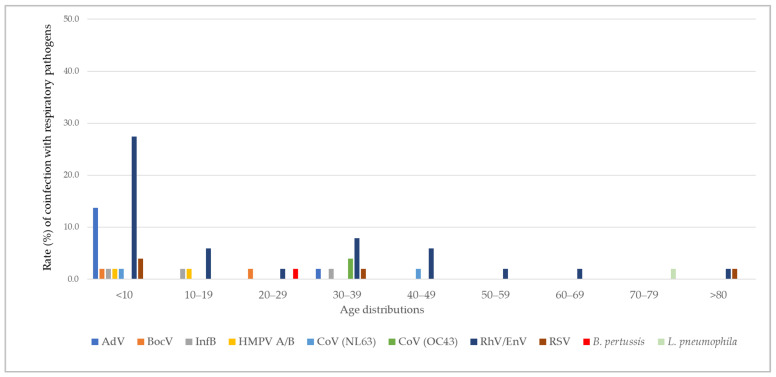
The distribution of respiratory viruses and bacteria by age group in NPS samples positive for SARS-CoV-2 and co-infections (*n* = 51) among the study subjects.

The variation in co-infection with age group can be seen in Figure 5. Among the co-infections with a single respiratory virus, rhinovirus/enterovirus (27.5%) and adenovirus (13.7%) were most detected in the <10 age group, followed by RSV (3.9%), bocavirus, InflB, HMPV A/B, and coronavirus NL63 (all at 2%). Similarly, the rate of co-infection with rhinovirus/enterovirus was highest in age groups 10–19 (5.9%), 30–39 (7.8%), 40–49 (5.9%), 20–29 (2.0%), 50–59 (2.0%), 60–69 (2.0%), and >80 (2.0%). Co-infection with two respiratory viral pathogens other than SARS-CoV-2 was detected in one sample in the age group 30–39 (coronavirus OC43 and RSV). Similarly, one sample in the age group 50–59 was found to be positive for coronavirus NL63 and rhinovirus/enterovirus. *B. pertussis* was detected as the co-infected bacteria in one sample in those aged 20–29 (2.0%), whereas *L. pneumophila* was detected in one sample in the age group 70–79 (2.0%).

## 4. Discussion

The COVID-19 pandemic, driven by SARS-CoV-2, has had a significant impact on global public health and the economy. Undoubtably, this pandemic has tested the resilience of health services across the globe and further exposed profound implications for health, economic progress, trust in governments, and social cohesions. The analysis presented here is a study of the rate of co-infections with respiratory pathogens in SARS-CoV-2-positive NPS samples in Cyprus. Successful completion of the project relied on the use of the QIAstat-Dx^®^ Respiratory SARS-CoV-2 Panel, reported for its robustness in the detection of respiratory pathogens [18,20]. To the best of our knowledge, this work is the first of its kind in the Cypriot population.

Based on systematic reviews and meta-analysis, viral coinfection and bacterial coinfection in SARS-CoV-2-infected patients are reported at 3–10% and 7–8% worldwide, respectively [9,10,11]. In Cyprus, data on SARS-CoV-2 co-infections are currently unavailable. However, a limited number of studies from geographically proximate countries have reported SARS-CoV-2 co-infections. For example, a study by Garazzino et al. in Italy reported viral co-infections in 6% of SARS-CoV-2-positive patients [21], a rate comparable to that observed in our study (4.5%). Bacterial co-infections in our study were detected in 0.1% of cases, which falls within the range reported in similar studies. In Italy, for instance, bacterial co-infection rates have been reported to range from 0.5% to 3% [21,22,23], while in Turkey, no bacterial co-infections (0%) were observed [24], and in Egypt, the rate was reported at 14% [25].

Currently, it remains unclear whether co-infections with SARS-CoV-2 and other viruses are less common than bacterial co-infections, with some—but not all—studies supporting this trend. This discrepancy may be partly attributed to differences in experimental design and analytical approaches, particularly the inclusion criteria for ICU and non-ICU patients, as reported in most studies [9,10,11]. Furthermore, data on co-infections among outpatients remain scarce, highlighting a gap in our understanding of the full spectrum of SARS-CoV-2 co-infections [13,14].

Influenza viruses are well-documented respiratory pathogens frequently detected alongside SARS-CoV-2 in numerous studies globally, with similar symptoms shared between these viruses. Reported co-infection rates vary widely, ranging from 0.08% to 52% across different geographical locations for influenza A [26,27,28,29], with influenza B co-infection rates ranging from 0% to 0.2% [30,31,32]. In our study, no samples were found to be positive for influenza A, whereas influenza B was detected in 0.3% of SARS-CoV-2-affected individuals. The reported incidence of co-infection with influenza A is slightly higher than that of influenza B among patients with positive diagnosis of SARS-CoV-2, possibly attributable to multiple factors, including the intrinsic viral properties of influenza A, seasonal epidemiological patterns, and differences in immune responses due to prior exposures [33]. The reason for the absence of influenza A detection in our study remains unclear; however, it may be partially attributable to the low number of co-infected samples analyzed and the fact that the dataset was not exclusively composed of hospitalized patients, where co-infection rates are often higher [30,33].

Our study found that rhinovirus/enterovirus was the most prevalent virus, accounting for 2.5% of co-infections, followed by adenovirus (0.7%) and RSV (0.4%). Rhinoviruses and enteroviruses are significant pathogens for humans and are the subject of intensive clinical and epidemiological research and public health measures [34]. Other similar studies have reported conflicting results, with some but not all studies showing rhinovirus/enterovirus to be the most prevalent virus in SARS-CoV-2-infected patients [26,35,36]. Due to the absence of SARS-CoV-2 variant typing in our dataset, it was not possible to examine whether specific viral strains were associated with distinct patterns of co-infection. Incorporating such analyses in future studies could help clarify potential interactions between SARS-CoV-2 variants and co-infecting respiratory viruses, providing a more detailed understanding of their epidemiological and clinical implications.

Previous studies have suggested that age is a key determinant in the likelihood of SARS-CoV-2 co-infections [37,38]. In line with these findings, our study observed the highest incidence of co-infections in the <10 age group, suggesting that younger age may be a potential risk factor for co-infections. Among these cases, rhinovirus/enterovirus was the most frequently detected co-infecting virus, accounting for 27.5% of cases, followed by adenovirus at 13.7% and RSV at 3.9%. Several factors may contribute to the increased incidence of co-infections in young children. Their distinct behavioral and social patterns, such as frequent close contact in day-care and kindergarten settings, create an environment conducive to viral transmission. Additionally, young children often have difficulty adhering to hygiene practices, such as wearing masks and regular handwashing, further increasing their risk of exposure to multiple pathogens. Moreover, their immature immune system compared to that of adults may render them more susceptible to simultaneous infections [39]. Interestingly, several infections seemed to rebound when non-pharmaceutical interventions were relaxed, resulting in an increase in co-infection rates [40,41]. In the present study, no significant trend was observed; however, a marginal increase in co-infection rates between 2020 and 2022 was noted. Expanding the sample size in future studies could enhance the robustness of these findings and allow for a more detailed analysis of co-infection patterns with individual pathogens.

A systematic review and meta-analysis conducted by Soltani et al. reported a pooled prevalence of bacterial co-infections of approximately 20% [12]. However, individual studies demonstrated a wide range of co-infection rates, varying from 0% [35] to 100% [42,43,44]. This considerable variability may be attributable to differences in study design, diagnostic methodologies, and clinical settings, as well as the widespread use of antibiotics, which can influence the detection and reporting of bacterial pathogens. In our study, bacterial co-infection was detected in 0.2% of SARS-CoV-2-positive samples, with *Legionella pneumophila* and *Bordetella pertussis* being the only identified bacterial pathogens. Currently, there is no consensus on the most commonly detected bacteria in SARS-CoV-2 co-infections. Reported bacterial co-pathogens vary across studies and include *Mycoplasma* species, *Haemophilus influenzae*, *Pseudomonas aeruginosa*, *Bordetella* spp., *Staphylococcus aureus*, and methicillin-resistant *Staphylococcus aureus* (MRSA) [12,45,46,47]. These discrepancies may be attributable to differences in study design, including variations in diagnostic methodologies, sample selection criteria, and patient populations analyzed [48]. Standardized approaches are needed to better characterize the prevalence and clinical significance of bacterial co-infections in SARS-CoV-2 cases.

The results presented in this study should be interpreted in light of its limitations. Firstly, sample collection was based on specimens submitted to the Molecular Virology Department for diagnostic purposes, without access to the participants’ medical history, clinical status, or treatment information. Consequently, categorizing individuals based on disease severity, comorbidities, or therapeutic interventions, factors that could significantly influence the risk and clinical impact of co-infections, was not possible. Although the value of such analyses in understanding the broader implications of co-infections is fully recognized, this limitation constrained the study’s ability to investigate potential risk factors or identify predictors of co-infection. Of equal importance, direct comparisons of the results presented herein with other studies should be carried out with caution, as most investigations on viral and bacterial co-infections in SARS-CoV-2-positive individuals have been conducted on hospitalized patients [9]. In addition, it must be acknowledged that co-infection and co-morbidity rates are typically higher in hospitalized populations, and this may lead to an underestimation of the prevalence in our study cohort of non-hospitalized patients [9]. Notably, only a limited number of studies have examined co-infections in outpatient settings [13,14]. Secondly, the number of SARS-CoV-2-positive samples analyzed for co-infections represented only 2% of the total available SARS-CoV-2-positive samples. This limitation was primarily due to financial constraints that could not be overcome. A larger sample size would have provided a more comprehensive understanding of co-infection patterns among SARS-CoV-2-infected individuals. Lastly, the absence of medical records for the referred patients restricted further analysis of potential associations between co-infections and clinical symptoms. Additionally, the lack of information regarding participants’ influenza vaccination status represents another limitation. Recent studies have shown that influenza vaccination can reduce the risk of influenza disease by 40–60% in the general population [49,50], highlighting its potential impact on co-infection rates. Such data could have been particularly valuable in interpreting the absence of influenza A detection in our cohort. A further possible explanation for the absence of influenza A detection could be the nature of the sampled population, which consisted of non-hospitalized individuals. Additionally, public health measures implemented to curb SARS-CoV-2 transmission—such as social distancing, mask mandates, and travel restrictions—may have significantly reduced influenza incidence and impact during the COVID-19 pandemic, as previously reported [51]. Indeed, Cyprus is one of the countries least impacted by the COVID-19 pandemic, as response measures were taken up early on during the pandemic.

## 5. Conclusions

This study provides previously unavailable data on viral and bacterial co-infections in SARS-CoV-2-infected individuals in Cyprus. Such studies are essential, as co-infections—particularly in outpatients—may facilitate the transmission of respiratory pathogens to various population groups, including vulnerable individuals such as children and the elderly. In addition, the identification of rhinovirus/enterovirus and adenovirus as the most prevalent co-infecting viruses in our cohort, along with the detection of rare bacterial pathogens, underscores the need for broad diagnostic screening during surveillance for respiratory infections. Equally important, continuous surveillance of multiple respiratory pathogens, especially in the context of evolving viral pandemics, remains a critical component of public health preparedness, as co-infections pose a significant challenge to healthcare systems worldwide, impacting both disease management and public health strategies. Future studies with larger sample sizes and access to clinical data will be necessary to better understand the impact of co-infections and to identify risk factors across different population groups and viral variant periods.

## Figures and Tables

**Table 1:** Demographic characteristics of study participants.

Variable	*n* = 1111	%
Gender		
Male	518	46.6
Female	593	53.4
Age		
<10	239	23.9
10–19	165	16.5
20–29	145	14.5
30–39	170	17.0
40–49	144	14.4
50–59	112	11.2
60–69	65	6.5
70–79	43	4.3
>80	28	2.8
Year		
March 2020–December 2020	381	34.2
January 2021–December 2021	555	50.0
January 2022–May 2022	175	15.8

**Table 2:** Frequency of co-infections with other respiratory pathogens in SARS-CoV-2-positive NPS samples during 2020–2022. NPS: nasopharyngeal swab.

Year	NPS *n* (%)	NPS + ve for One Other Virus *n* (%)	NPS + ve for Two Other Viruses *n* (%)	NPS + ve for Bacteria *n* (%)
2020	381 (34.3)	12 (3.1)	0 (0)	2 (0.5)
2021	555 (49.9)	25 (4.5)	2 (0.4)	0 (0)
2022	175 (15.8)	10 (5.7)	0 (0)	0 (0)
Total	1111	47 (4.1)	2 (0.2)	2 (0.2)

**Table 3:** Frequency of co-infections with other viruses and bacteria in SARS-CoV-2-positive NPS samples during 2020–2022. RhV: rhinovirus; EnV: enterovirus; HMPV A/B: human metapneumovirus A/B; AdV: adenovirus; CoV: coronavirus; InfB: influenza B; RSV: respiratory syncytial virus; BocV: bocavirus; *LP*: *Legionella pneumophilia*; *BP*: *Bordetella pertussis*.

Year	RhV/EnV *n* (%)	HMPV A/B *n* (%)	AdV *n* (%)	CoV (NL63) *n* (%)	CoV (OC43) *n* (%)	InfB *n* (%)	RSV *n* (%)	BocV *n* (%)	*LP* *n* (%)	*BP* *n* (%)
2020	9 (2.4)	0 (0)	2 (0.5)	0 (0)	0 (0)	0 (0)	1 (0.3)	0 (0)	1 (0.3)	1 (0.3)
2021	14 (2.5)	0 (0)	3 (0.5)	2 (0.4)	2 (0.4)	3 (0.5)	3 (0.5)	2 (0.4)	0 (0)	0 (0)
2022	5 (2.9)	2 (1.1)	3 (1.7)	0 (0)	0 (0)	0 (0)	0 (0)	0 (0)	0 (0)	0 (0)
Total	28 (2.5)	2 (0.2)	8 (0.7)	2 (0.2)	2 (0.2)	3 (0.3)	4 (0.4)	2 (0.2)	1 (0.1)	1 (0.1)

## Data Availability

The original contributions presented in this study are included in the article. Further inquiries can be directed to the corresponding author.

## Abbreviations

The following abbreviations are used in this manuscript:

NPS	Nasopharyngeal swab
RhV	Rhinovirus
EnV	Enterovirus
HMPV A/B	Human metapneumovirus A/B
CoV	Coronavirus
BocV	Bocavirus
LP	*Legionella pneumophilia*
BP	*Bordetella pertussis*
ICU	*Intensive care unit*
